# Medical History of Texas

**Published:** 1915-11

**Authors:** Hugh W. Crouse

**Affiliations:** El Paso, Texas


### Medical History of Texas.

The following letter from Dr. Crouse should meet with the hearty co-operation of those who are interested in preserving the medical history of Texas. It is a splendid work. Please communicate with the doctor at once, and if you can assist him in any way he will appreciate it very much.—Ed.

El Paso, Texas, October, 1915.

Dear Mrs. Daniel: I have been trying to gather some of the medical history of Texas, including anecdotes, letters, pamphlets, books, old instruments, pictures, and photos of men and places, but my search hitherto has not been very successful, for much of the human side of our old doctors has died with the men who remembered the stories concerning them.

There were medical men in Texas who did good work otherwise than in medicine—geology, botany, etc.—but the record, not the lasting good, is buried in a book or pamphlet long out of print.

If anyone would lend me material I would guard it carefully and promptly return it, and I would urge every State and county medical society to form a collection of things appertaining to the past and let them be the nucleus of a section in the town museum. Curiously, it is the relatives of a man who can tell least concerning his medical work or professional life.

Faithfully yours,

HUGH W. CROUSE.

